# Preclinical and translational studies of fenobam, an mGlu5 NAM, for the treatment of pain

**DOI:** 10.1186/1744-8069-10-S1-O3

**Published:** 2014-12-15

**Authors:** Robert W Gereau IV, Laura F Cavallone

**Affiliations:** 1 Washington University Pain Center and Department of Anesthesiology, Washington University School of Medicine, St Louis, MO 63110, USA

## Background

Metabotropic glutamate receptor 5 has been suggested by many rodent studies to be a promising target for the development of novel analgesic drugs. The lack of approved compounds has prevented proof-of-concept studies in human subjects. Here we describe preclinical and translational studies of the mGlu5 negative allosteric modulator (NAM), fenobam.

## Materials and methods

Fenobam was tested for analgesic efficacy and toxicity in mouse models. We also tested the plasma levels after oral dosing of fenobam in healthy volunteers, and collected any adverse events following oral dosing compared to placebo.

## Results

The mGlu5 NAM Fenobam is effective in a wide variety of preclinical pain models in mice with no evidence of the development of analgesic tolerance on daily dosing. No obvious toxicities were observed in mice, or in several studies in healthy human volunteers.

## Conclusions

Fenobam has robust analgesic activity and shows a good safety profile. Fenobam therefore represents a useful tool for proof-of-concept studies in human subjects.

